# COVID-19 patients exhibit less pronounced immune suppression compared with bacterial septic shock patients

**DOI:** 10.1186/s13054-020-02896-5

**Published:** 2020-05-26

**Authors:** Matthijs Kox, Tim Frenzel, Jeroen Schouten, Frank L. van de Veerdonk, Hans J. P. M. Koenen, Peter Pickkers, , on behalf of the RCI-COVID-19 study group, Pleun Hemelaar, Remi Beunders, Johannes van der Hoeven, Sjef van der Velde, Hetty van der Eng, Noortje Rovers, Margreet Klop-Riehl, Jelle Gerretsen, Emma Kooistra, Nicole Waalders, Wout Claassen, Hidde Heesakkers, Tirsa van Schaik, Mihai Netea, Leo Joosten, Nico Janssen, Inge Grondman, Aline de Nooijer, Quirijn de Mast, Martin Jaeger, Ilse Kouijzer, Helga Dijkstra, Heidi Lemmers, Reinout van Crevel, Josephine van de Maat, Gerine Nijman, Simone Moorlag, Esther Taks, Priya Debisarun, Heiman Wertheim, Joost Hopman, Janette Rahamat-Langendoen, Chantal Bleeker-Rovers, Esther Fasse, Esther van Rijssen, Manon Kolkman, Bram van Cranenbroek, Ruben Smeets, Irma Joosten

**Affiliations:** 1 Department of Intensive Care Medicine, Radboud University Medical Center, Internal Mail 710, PO Box 9101, 6500 HB Nijmegen, The Netherlands; 2 Radboud Center for Infectious Diseases (RCI), Radboud University Medical Center, Nijmegen, The Netherlands; 3 Department of Internal Medicine, Radboud university medical center, Nijmegen, The Netherlands; 4 Department of Medical Immunology, Radboud university medical center, Nijmegen, The Netherlands

**Keywords:** HLA-DR, mHLA-DR, COVID, COVID-19, SARS-CoV-2, Monocytes, Immune suppression

Low monocytic (m)HLA-DR expression is the most widely used marker of innate immune suppression in critically ill patients. We recently showed that in bacterial septic shock patients, low mHLA-DR expression is prevalent and associated with the development of secondary infections [1]. At the end of March 2020, there were in excess of 800,000 confirmed cases of coronavirus disease 2019 (COVID-19) worldwide, of whom more than 12,000 from the Netherlands. Several reports suggest that patients with severe COVID-19 may suffer from a hyperinflammatory “cytokine storm” [2, 3]. However, unlike SARS-CoV infection, high levels of anti-inflammatory mediators (e.g. IL-10 and IL-4) have also been reported in COVID-19 [3]. Although there are few indications that secondary infections are common in COVID-19 patients, one study reported that 16% of COVID-19 patients who died developed secondary infections [4], which might indicate an immune-suppressed state. Herein, we explored mHLA-DR expression kinetics in a cohort of 24 critically ill COVID-19 patients.

Between March 18 and 27, all COVID-19 patients admitted to our intensive care unit (ICU) were included in this prospective observational study. COVID-19 was confirmed by two positive RT-PCR tests for SARS-CoV-2 in throat swabs and by CT scan findings. Fourteen patients were transferred from other ICUs. The median ICU length of stay at the time of study inclusion was 3 days. The study was carried out in accordance with the applicable rules concerning the review of research ethics committees and informed consent in the Netherlands. All patients or legal representatives were informed about the study details and allowed to abstain from participation. Ethylenediaminetetraacetic acid (EDTA)-anticoagulated blood was stored at 4–8 °C until mHLA-DR expression analysis (performed within 2 h after withdrawal). Expression levels were determined using the Anti-HLA-DR/Anti-Monocyte Quantibrite assay (BD Biosciences, San Jose, USA) on a Navios flow cytometer and software (Beckman Coulter, Brea, USA). Total number of antibodies bound per cell (mAb/cell) were quantified using a standard curve constructed with Quantibrite phycoerythrin beads (BD Biosciences). All other data were extracted from the electronic patient record. For patients who were transferred from other ICUs, patient characteristics were obtained at admission to our ICU. Data were analysed using SPSS Statistics v22 (IBM, Armonk, USA) and GraphPad Prism v8.3.0 (GraphPad Software, La Jolla, USA).

Patient characteristics are listed in Table 1. In line with previous observations [3], the majority of patients was male and many had comorbidities. The median time from onset of COVID-19 symptoms to ICU admission was 11 days. All patients were mechanically ventilated and exhibited increases in inflammatory parameters (Table 1). As of March 27, 2020, two patients died (at 3 and 4 days post-ICU admission, data of only one timepoint of these patients was recorded), and 22 patients were still in the ICU.

**Table 1:** Patient characteristics

Characteristics	All patients (***n*** = 24)
Age, years	69 [61–73]
Sex	
Female	6 (25%)
Male	18 (75%)
Body mass index, kg/m^2^	27.5 [24.3–31.1]
Any comorbidities	19 (79%)
Diabetes	7 (29%)
Hypertension	6 (25%)
Cardiovascular disease	7 (29%)
Chronic obstructive pulmonary disease	3 (13%)
Malignancy	10 (42%)
Chronic liver disease	0 (0%)
Chronic kidney disease	1 (4%)
Immunocompromised*	5 (17%)
APACHE II	17 [11–21]
Time from illness onset to ICU admission, days	11 [8–13]
**Medication use**	
Norepinephrine use	20 (83%)
Maximum infusion rate in first 24 h on ICU, μg/kg/min	0.11 [0.07–0.21]
Corticosteroids	1 (4%)
Remdesivir	3 (13%)
Chloroquine	19 (79%)
Anakinra	1 (4%)
**Symptoms and (laboratory) parameters**	
Heart rate, bpm	83 [71–112]
Mean arterial pressure, mmHg	77 [72–81]
Fluid balance in first 24 h on ICU, mL	1348 [680–1881]
Urine output in first 24 h on ICU, mL	1105 [888–1486]
Creatinine, μmol/L	86 [70–133]
Dialysis	0 (0%)
Mechanical ventilation (invasive)	24 (100%)
Tidal volume, mL/kg	5.3 [4.4–6.0]
Respiratory rate, bpm	21 [20–24]
PEEP, cm H_2_O	12 [10–14]
FiO_2_, %	50 [41–60]
P/F ratio	164 [136–189]
100–200	20 (83%)
200–300	4 (17%)
Thrombocytes, 10^9^/L	239 [151–274]
Leukocytes, 10^9^/L	8.2 [5.3–11.6]
C-reactive protein, mg/L	301 [157–316]
Procalcitonin, μg/L	0.72 [0.29–3.66]
Ferritin, μg/L	1216 [488–1834]
Lactate (highest over last 24 h), mmol/L	1.2 [1.1–1.7]
D-dimer, ng/mL	3075 [1780–4598]
Troponin I, ng/L	23 [13–44]
Albumin, g/L	20 [17–22]
Alanine aminotransferase, U/L	34 [21–41]
Aspartate aminotransferase, U/L	48 [31–73]
Creatinine kinase, U/L	136 [56–357]
Lactate dehydrogenase, U/L	398 [303–499]
**Outcome parameters**	
Secondary infections	0 (0%)
Death	2 (8%)

Data were obtained at study inclusion and are presented as *n* (%) or median [IQR]

*Chronic use of immunosuppressive medication

Although mHLA-DR expression levels in COVID-19 patients were lower than those observed in healthy subjects (15,000–45,000 mAb/cell [5]), the extent of suppression was less pronounced than observed in bacterial septic shock patients (geometric mean [95% CI] of 11,860 [11,035–12,746] vs. 5211 [4904–5537] mAb/cell, respectively; *p* < 0.0001; Fig. 1a, sepsis data from [1]). mHLA-DR expression kinetics revealed no change over time (Fig. 1b). Circulating C-reactive protein concentrations declined over time (Fig. 1c), whereas no significant changes in circulating procalcitonin, leukocytes, or ferritin levels were observed (Fig. 1d–f). None of the patients developed a secondary infection during the follow-up period (last recorded timepoint: 16-17 days post-ICU admission, see Fig. 1).

**Fig. 1:**
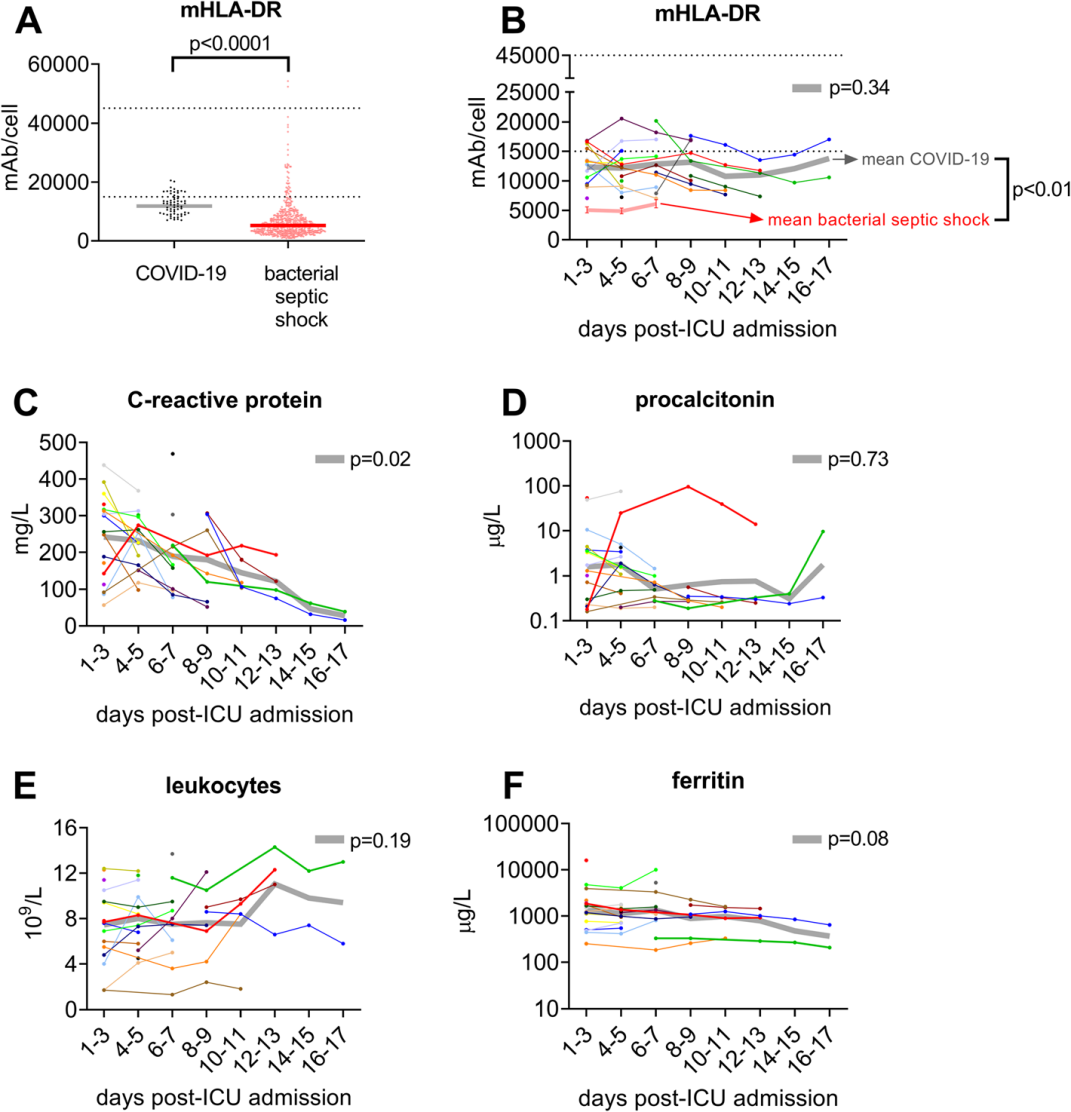
**a** mHLA-DR expression in patients with COVID-19 (*n* = 24, multiple timepoints) and bacterial septic shock (*n* = 241, days 1–2, 3–4, and/or 6–8 after onset of septic shock, obtained using the same methodology, data recently published [1]). Horizontal line indicates geometric mean. The dotted lines indicate the reference range in healthy subjects [5]. *p* value calculated using unpaired *t* test on log-transformed data. **b**–**f** Kinetics of mHLA-DR expression, circulating C-reactive protein, procalcitonin, leukocyte numbers, and ferritin in COVID-19 patients (individual data are shown, *n* = 24). The transparent grey line represents mean (**b**, **c**, **e**) or geometric mean (**c**, **e**) values of the entire cohort. The transparent pink line in **b** represents data obtained from [1] (geometric mean ± 95% CI, please note that values obtained at days 1–2 (*n* = 203), 3–4 (*n* = 205), and 6–8 (*n* = 133) after onset of septic shock are plotted at days 1–3, 4–5, and 6–7, respectively). The dotted lines in **b** indicate the reference range in healthy subjects [5]. *p* values next to the transparent grey line represent changes over time in COVID-19 patients, calculated using mixed model analysis (on log-transformed data for **d** and **f**). Differences between COVID-19 and sepsis patients in **b** were analysed using unpaired *t* tests on log-transformed data (*p* < 0.0001 on days 1–3 and 4–5, and *p* = 0.0015 on days 6–7)

In conclusion, despite a pronounced inflammatory response in COVID-19 patients, our preliminary results indicate more moderate innate immune suppression compared with bacterial septic shock patients. These findings are in accordance with a low incidence of secondary infections in COVID-19 patients. Therefore, innate immune suppression as a negative feedback mechanism following pathogen-associated molecular pattern-induced inflammation appears less pronounced in COVID-19.

## Data Availability

All data generated or analysed during this study are included in this published article.
